# Acknowledgement to reviewers

**DOI:** 10.1530/EO-6-A1

**Published:** 2026-01-19

The editorial board wishes to thank the following individuals who have helped to review manuscripts for *Endocrine Oncology* during 2025. Their assistance is gratefully acknowledged.

**Table:** 

S Adam
S Agarwal
S Agosto
A Akirov
O Alagoz
Ö Alan
M Albertelli
K I Alexandraki
A Aljubran
J Aller
S Alli
H Almeamar
L O Almeida
B Altieri
C V Alvarez
C Alvarez-Escola
M Alves
V Ambrosini
E Anda Apiñaniz
L Andereggen
M Andreassen
T E Angell
A Angelousi
L Annaratone
D Annibali
L Apostolidis
N Arandi
D Arangalage
M Araujo-Castro
A Arjona
C Artigas
P Arumugam
A K Arveschoug
M Atiquzzaman
M Auer
R Axelsson
J Ayuk

J H Baek
C Bal
O Balague
S Baldari
C Baldwin
G I Baltatescu
L Bandeira
M Barnett
I Bartolini
L R Barzaghi
B Baussart
J-M Beauregard
V L Beesley
A Belfiore
J Bell
A Belli
Z Benbrahim
J Berardinelli
E K Bergsland
M C Berkovic
A Berruti
F Beuschlein
C L Bevan
M Bevere
M Bhandare
K Bhaskaran
A B H Bhatti
B Biagetti
N R Biermasz
A Binnetoglu
M W Bishop
L Blomqvist
L Bodei
E Boehm
U Boggi
M Bondanelli
A Bongiovanni
I Borbath
M Borowczyk
M D Bortz
L Boucai
C Bouvier
M F Bozkurt
A J A T Braat
T Brabander
S Braber
R Brady
P Breheny
J M Briceno
N C Brownstein
T Brue
M Brunotto
A Buffet
M J Bugalho
D Bulzico
P Burman
M Burnier
M G Burt
J R Buscombe

M E Cabanillas
B Cakmakoglu
A Canella
L Canu
C Capatina
J Capdevila
M Caplin
D Capoccia
D Cardinale
C Carlberg
F E Carr
J A Carrasquillo
R Carroll
R T Casey
P H Cashin
M Castellana
L A Castroneves
C Cattaneo
J Cernich
S Chakrabarti
B G Challis
R Chammas
A P Chan
D Chan
J A Chan
A Chang
Y Che
L Chen
Y Chen
Z Chen
T R Chesney
L Chiavaroli
E Christ
M Christ-Crain
S Cingarlini
N A Cipriani
C Cittanti
M Cives
T E Clancy
C N Clarke
K Colston
K W Colston
A Conzo
E C Coopmans
A Coppola
F Cordido
C Correa-Gallego
A-S Cottereau
V Cracolici
D Cronin-Fenton
T Cuny
G Curigliano

A M da Silva Moreira Francisco
A F Daly
G Dam
L Das
K Daskalakis
E De Azambuja
W W de Herder
A De Jesus-Acosta
A De la Vieja
J M de Laat
M del Rio-Moreno
J Del Rivero
J Deleemans
T Denecke
C Deroose
E Deshayes
T Dhaouadi
G Di Dalmazi
G Di Perna
L O Dierickx
J J Díez
U Dischinger
L Docimo
M Dottermusch
B Drake
H Dralle
M Druce
M R Druce
A Durand
A Dzikiewicz-Krawczyk

A Ebrahimi
A Eketunde
I Ellis
T Else
R Elshazli
M Escolino
D Eskandari
F Esteso
J Evron
S Ezzat

A Faggiano
J A Fagin
M Falconi
G Fanciulli
P A Fasching
M Fassnacht
D Feldman
E Fennell
A Fernández-Pombo
D Ferone
V Fiorentino
C Fiorillo
J Fitzgibbon
M Fleseriu
J Flitsch
A Fodor
L Fozzatti
M C Fragoso
O Franklin
S Franks
F Frasca
C Fribbens
A Frilling
W Frishman
L Fugazzola
F Fujishima
V Fusco

F Gabalec
F Gaia
M Gallo
J C Galofré
F Gatto
P R Gazzola Zen
J Gebauer
H Geijer
F Gelsomino
L Gerard
I Gesmundo
S Ghaem-Maghami
L Gilardi
M Gild
A Gillis
M Giordano Imbroll
L Giovanella
A Glezer
A Glover
J Gómez Tejeda Zañudo
P Goodwin
E Goossens
R Graff
U Graham
C M Grana
G Grani
J Grasselli
Y Greenman
F Grillo
J J Grob
J C Groblewski
D Groener
M Grönberg
A B Grossman
C Grötzinger
S Grozinsky-Glasberg
E G Grubbs
T Grüning
R Guenter
S Guillén-López

M A Habra
M Haciyanli
R Haddad
J Hadoux
M Haissaguerre
R Hakkak
T R Halfdanarson
J Hallet
E Y Hanna
C Happel
I Harbuz-Miller
M Hasosah
A Hawazie
A Hayes
H He
N He
S Hecht
A Heck
J Heldin
S Higuchi
A L Ho
A O Hoff
J Hofland
L Hofland
L J Hofland
P Holmager
S J Hong
R H Hope
Y Horimoto
N Hossain
D Howell
M I Hu
W Y Hu
I Hussain
M Hussein

J Idkowiak
K Idres
P Iglesias
A Imperiale
W J Inder
K Insogna
A G Ioachimescu
M V Iorio
E Isenberg-Grzeda
L Izatt

A Jacob
M J Jager
A R Janecke
M A Jara
F J Jenkins
T Jentzsch
J Jiang
P Jimenez
M Jordà
C B Juhl
A Jukes
M Justyna Kozieł

P Kadioglu
G Kaltsas
G A Kaltsas
G Kamiński
E Kandil
N Karavitaki
B Karbassi
I Karfis
A Kargi
S Kaslow
D Kastelan
I Kastrati
L Kasuki
D T Katz
M Kawai
A T Kendi
H K Khan
M Khan
B Kiesewetter
W G Kim
J King
K Kiseljak-Vassiliades
C Kitahara
A Kjær
J Kjaer
P Klener
G Klöppel
J Klubo-Gwiezdzinska
T Kolarova
G Kong
P Kongkham
B Konukiewitz
B S Koo
A L L F Kooi
M Koopaie
L S Kornerup
J Koshiol
A Kotwal
A Kowalska
J Krajewska
I Kreitschmann-Andermahr
S Krishnamurthy
M Kroiss
S Krug
I Y Krynytska
A Kula
K Kusnierz
J Kwekkeboom
G Kyriakopoulos

A Lacroix
M Laganà
A Lamarca
E Lamback
M L Lamers
L-S Landwehr
B Lang
S W Langer
A G A Lania
T Lanisnik Rizner
A D Le
M Lechner
M LeCompte
B Lecumberri Santamaria
A Y Lee
E L Lee
S Lee
C Lepage
I Leung
M Li
S K Libutti
C T Lim
E Lim
K H J Lim
B Lindeman
K E Lines
L Lipscombe
T S Lisse
C Liu
M J Livhits
J J Loonen
A Lopez-Aguiar
J P P Low
M Luconi
P M Lykoudis

J Macfarlane
Y M Machlah
R M B Maciel
S Madhunapantula
C Maenhaut
A Mahamid
A L Maia
P Malandrino
A Malczewska
G Malhotra
A Mamelak
J Mangion
A Maniakas
W Mansoor
T Mansur
S Mao
M Marazuela
U Marchese
H Marcus
F Marini
Z O Martinez
D Martorana
S Massironi
A Mathew
P Matos
A Matrone
K Matsumoto
S Maurea
H Mazeh
G Mazziotti
M Mazzola
A I McCormack
B McGowan
T McVeigh
P Mercantini
J F Merino-Torres
O Mete
R Mikolajczak
M Mileva
M Milione
I M Min
E Minna
A L Mitchell
R Modica
S Montoto
L Morelli
B Moretti
D L Morganstein
K Mortezaee
O Mosalem
S Mukherjee
A Munir
J L Muñoz de Nova
A Munteanu
N Murphy
R D Murray
J Muto

Y Nagashima
K Nakagawa
Y Nakamura
N W Nalruwaili
K Nanba
M Nannini
B Naraev
I Nassour
S Navalkissoor
J-T Navarro
P Navin
L Nedar
R T Netea-Maier
K Newbold
P J Newey
N Q Nguyen
L Ni
G P Nicolas
A Nieri
A Niessen
E J Nieveen van Dijkum
Y E Nikiforov
M N Nikiforova
A V Nikitski
J E Noel
S Nölting
E Nordenström
N Nyilas

K E Oberg
J M OConnor
Y Oda
N P Ohori
T Okamoto
I Olesrud
K M Oliveira
K Ono
M Ooms
N Oriuchi
S O'Toole
P S Oturai
J A Overbeek

K Pacak
G Paganelli
G Palmini
R Paluri
C Pamporaki
R B Panchangam
K Pantino
M Papotti
I Paredes
S Parisien LaSalle
A Patrizio
R Patrone
D A Pattison
M E Pavel
E Pelle
G Pellegriti
A Pera-Rojas
A Perathoner
F Pereira
A Peri
J Perinel
A Perren
P Petrossians
C Petry
C E Pierreux
E M Pinto
F Pitoia
R Pollack
M N Pollak
J Pozniak
S R Prasad
V Prasad
F Primavesi
A Prinzi
T Prolla
M Puig-Domingo

G Quero

M Radan
A R Raimondi
J Ramage
R A Ramirez
E R Ramos Figueira
S N Rao
F Raue
S Ravaioli
G Raverot
D Rayson
N S Reed
J Refardt
J Rege
R Reis
F Ren
Y Ren
C Rentsch
K E Rentscher
N Renwick
J C Ricarte-Filho
R H Rice
S Richter
K Ricklund
R P Riechelmann
G Riesco-Eizaguirre
M D Ringel
J M M Rogasch
P Romanet
C L Ronchi
F Rosch
E Rossi
R Rotermund
M Roy
E Royo
M Ruchala
R M Ruggeri
B M Ryan

S M Sadowski
J Sahoo
M Saitou
S Saji
L Salvatore
A Sanabria
C Sanchez-Ragnarsson
P Santisteban
V Sarathi
S Sbiera
N Schaefer
G Schembri
J Schrader
J Scianowski
R-D Seban
Y Seimbille
F Sellner
H Seppänen
M Serebro
J Serrano-Lopez
G Sesti
Y Sethi
S Severi
M Shakibaei
K M Shekhda
M Sherlock
S Sherman
Y Shi
I Shimon
P Simonen
M Sinn
A Skalniak
B Skogseid
M Skok
A V Snezhkina
H P Soares
M H Son
M B Sonbol
H Sorbye
I C Sormaz
J Soukup
A Sowa-Staszczak
F Spada
I Sperduti
C Spitzweg
D Sprengers
R Srirajaskanthan
S Staettner
P Stålberg
M Stasiak
L J States
S E Steck
C Stewart
C A Stratakis
J R Strosberg
I Sugitani
T Sugiyama
Y Sun
K E Sundling
S Swarnakar
E Świętochowska

A Tabarin
R Taboada
M Tacelli
D Taieb
H Takahashi
R Tamimi
G Tapia
G Targher
K Tarte
R A Taylor
F Tenenbaum
R Tenta
M Terzolo
M E T Tesselaar
M Tewari
A Thakar
R V Thakker
S Theurer
R Thomsen
H J L M Timmers
K C Tjioe
E Toledo
C W Tomas
L Torregrossa
G T Torrezan
C Toumpanakis
H Toyama
P Tralongo
T-V-T Tran
M Trevisan
N Tritos
D Tsang
M Tsoli
R P Tufano
D Turchetti
A Tyberg

S Uccella
N Unger
M Urbini
L Urso

P Valderrabano
G D Valk
T M van Ginhoven
E van Velsen
S Veldhuijzen van Zanten
I Velikyan
R Vergeer
J S P Vermaat
A Verrienti
C Verslype
A Verstuyf
A Villani
R Villar-Taibo
M M Vivanco
M Volante
F Völter
A Voogd

A Wahba
D Wang
J R Wang
W Wang
Z Wang
D Waniczek
J D Wasserman
A Wassner
M O Weickert
Y Weng
B Whitelaw
D T Wijeratne
B Winzeler
I Witterick
K Wolf

J Xiang
J Xiao
C Xing
M Xing
F Xiong
L Xiong

K Yamamoto
K Yamanoi
Y Ye

D E Zantut-Wittmann
R Zarnegar
J Zavadil
A Zhang
J Zhang
W Zhang
P Zheng
M Zhou
A Ziaee
C E Zurstrassen

